# Prevalence of Dental Caries and Fissure Sealants in the First Permanent Molars among Male Children in Riyadh, Kingdom of Saudi Arabia

**DOI:** 10.5005/jp-journals-10005-1541

**Published:** 2018-10-01

**Authors:** Mohammed S Aldossary, Abdullah A Alamri, Sattam A Alshiha, Mohammed A Hattan, Yazeed K Alfraih, Hessa M Alwayli

**Affiliations:** 1 Specialist in Pediatric Dentistry, Department of Dental Department, Ministry of Health, Riyadh, Kingdom of Saudi Arabia; 2 Consultant, Department of Dental Department, Ministry of Health, Riyadh, Kingdom of Saudi Arabia; 3 Consultant, Department of Dental Department, Ministry of Health, Riyadh, Kingdom of Saudi Arabia; 4 Specialist in Pediatric Dentistry, Department of Dental Department, Ministry of Health, Riyadh, Kingdom of Saudi Arabia; 5 General Dentist, Department of Dental Department, Ministry of Health, Riyadh, Kingdom of Saudi Arabia; 6 Consultant, Department of Dental Department, Ministry of Health, Riyadh, Kingdom of Saudi Arabia

**Keywords:** Cross-sectional survey, Dental caries, First permanent molar, Fissure sealants, Preventive dentistry.

## Abstract

**Aims:**

To evaluate the prevalence of dental caries and the presence of fissure sealants on the first permanent molars (FPMs) among 6 to 9 years old primary school boys in Riyadh, Kingdom of Saudi Arabia.

**Materials and methods:**

The carious status and the presence of fissure sealants on the FPMs were examined in 1844 schoolboys, aged 6-9 years (the first three grades), from 17 randomly selected primary schools in Riyadh city, Kingdom of Saudi Arabia.Chi-square test was used to assess the significance of differences in prevalence and proportions.

**Results:**

A total of 5394 FPMs were assessed in the 1844 children. Eighty-three point five percent of children were caries free. Only 0.8% of the children had at least one fissure sealant applied. At tooth level, the decayed FPMs counted for 10.6%. There was obvious underuse of fissure sealants on the FPMs; 0.5% (n = 25). The caries prevalence in the mandibular FPMs (14.4%), was significantly higher than in the maxillary FPMs, 7.7% (p < 0.01). The proportion of carious FPMs increased with age of the children significantly (p < 0.01). There was no significant difference in the proportion of the presence of fissure sealants among the three different grade/age groups (p > 0.05).

**Conclusion:**

Caries prevalence in the FPMs was moderate but serious amongst this cohort of young students. This was opposed with very low use of fissure sealants.

**Clinical significance:**

Careful examination of the first permanent molars among children is mandatory because of their high caries susceptibility. It is important to increase public and dental professionals’ awareness of the effectiveness of fissure sealants and encouraging more placement.

**How to cite this article:** Aldossary MS, Alamri AA, Alshiha SA, Hattan MA, Alfraih YK, Alwayli HM. Prevalence of Dental Caries and Fissure Sealants in the First Permanent Molars among Male Children in Riyadh, Kingdom Saudi Arabia. Int J Clin Pediatr Dent., 2018;11(5):365-370.

## INTRODUCTION

Dental caries is a serious public health problem worldwide. Although preventable, it is still considered as a chronic disease with high prevalence affecting many children in several countries including Kingdom of Saudi Arabia.^1-3^

Dental caries is a multifactorial disease involving various risk and preventive factors. In the permanent dentition, the first molar exhibits an increasedsuscepti-bility to caries in the occlusal pits and fissures due to its morphological characteristics, the early time of its eruption, and its positioning in the oral cavity.^4-6^

The (FPM) is important for the dentition and dental development, and early loss due to caries would have a significant impact on the future dental health of the child.^7^ In addition, this is considered as expensive, time-consuming and traumatic for the young child.^8^

Because of the higher frequency of caries on the FPM, with the occlusal surface remains the most prevalent of the carious area within a short period following its eruption, such surfaces would need to be sealed.^4,7,9,10^

In addition to other caries preventive measures and procedures, fissure sealants are recommended mainly on the permanent posterior teeth, acting by preventing the development and growth of plaque and bacteria in the grooves.^4,11-13^

There is evidence in the literature regarding fissure sealants’ effectiveness in caries prevention and control, for both individual and community-based interventions for children and adolescents.^4,9,12-15^

Oral health surveys provide an idea about the population’s oral health status, treatment needs, and provides baseline information for establishing oral health plans as per the actual needs of their population.^16^ In order to plan and establish the national preventive program with sealants in a community, it is mandatory to know the epidemiological pattern of caries, caries risk of individuals, and caries prevalence of the country.^4,17^ The school-based sealant preventive programs are highly recommended for their effectiveness.^18,19^

In Riyadh, the capital city of Saudi Arabia, there have been no studies undertaken to determine the prevalence of dental caries in the FPMs, and it is essential to obtain baseline data regarding their condition, so appropriate prevention and treatment needs can be implemented. For such a sealant preventive program, some knowledge regarding the existing usage of sealants to caries needs to be answered, to determine the feasibility and appropriateness of this type of intervention at a National level.

This cross-sectional survey aimed to evaluate the prevalence of dental caries and the presence of fissure seal-antson the FPMs among 6 to 9 years old primary school boys in Riyadh, Saudi Arabia. Moreover, to provide a foundation for the current caries prevention strategies.

## MATERIALS AND METHODS

### Population

This cross-sectional study was conducted from October 2015 to May 2016 at public male primary schools in Riyadh city, Saudi Arabia. It was carried out as a part of the annual dental preventive program targeting primary school children, organized by the Preventive Dentistry Department, Ministry of Health, Riyadh, Kingdom of Saudi Arabia. The program includes an educational part on oral hygiene and topical fluoride application. Ethical approval was obtained from the Preventive Dentistry Committee, Ministry of Health, Riyadh, Kingdom of Saudi Arabia.

A total of 17 public male primary schools were randomly selected from different five geographic locations within Riyadh city.

The schools were officially informed, and visitation permissions and coordination with the schools were obtained from the Ministry of Education and school principals to arrange a day for data collection. Written consents were obtained from children’s parents.

In this survey, 1,844 male students were examined, which included all children with an obtained consent form, in the first three grades within the selected school (first, second, and third grades). The age of school children at first grade is 6 to 7; second grade is 7 to 8, and third grade is 8 to 9 years old.

Before commencing the examination, a brief orientation and oral hygiene instructions to the students were carried out. Topical fluoride varnish (Clinpro^TM^ 5% Sodium Fluoride White Varnish, 3M ESPE, St. Paul, MN, USA) was applied following examination to the children with obtained informed consent.

### Screening

Oral examination was conducted by ten examiners (trained and calibrated male hygienists) who were divided into five teams. In this way, each team included two examiners; one either conducted examination or assisted in data entry in a designed data collection form.

The children were examined, in their classroom with the child sitting on a conventional; non-dental classroom chair, with a disposable mouth mirror, wooden tongue blade, and a probe, when needed, under adequate natural daylight, and under all infection control measures. The probe was used sparingly on doubtful surfaces. In case of any doubt, the tooth was marked sound.

For this study, only the FPMs of the children were recorded. The examination and record consist of all FPMs which the full occlusal surface of the crown exposed. Partially erupted or unerupted FPMs were excluded. Dental caries was assessed using the World Health Organization (WHO) diagnostic criteria for the oral health survey.16 The decayed FPMs were recorded as decayed regardless of the carious lesion stage. The fissure sealants were recorded as present or absent.Fissure sealant’s presence on the FPMs hasrecorded irrespective the status of the sealant whether intact or partially lost.No radiographs were taken.

### Statistical Analysis

Inter- and Intra-examiner reproducibility were assessed using weighted kappa statistics by re-examining a group of 50 children, four weeks after the start of the study. A very high degree of agreement was demonstrated (Kappa > 0.80).

All data were managed and edited using Microsoft^®^ Excel^®^ (Microsoft^®^ Office 2007, Microsoft^®^ Corp, Redmond, WA, USA). All statistical analyses were performed using Statistical Package for Social Sciences (IBM SPSS Statistics 20.0 Armonk, NY, USA). Descriptive statistics and frequencies were generated.

Chi-square test was used to assess the significance of differences in prevalence and proportions at tooth level. The level of statistical significance was set at α = 0.05.

Comparisons were made between grades (first, second, third) which reflect the age groups, and between maxillary versus mandibular FPMs.

## RESULTS

In the 1844 children examined, a total of 5394 FPMs were assessed for carious status, the presence of restoration, the presence of fissure sealants, or being sound. Partially erupted and unerupted FPMs were excluded (n = 1982).

None of the teeth was recorded as missing due to caries. The children who had at least one decayed or filled FPMs (i.e., caries prevalence) counted for 16.5% (n = 305 children). In other words, 83.5% of the children (n = 1539), did not have caries experience in their FPMs (i.e., caries free). Only 0.8% of the children (n = 15), had at least one fissure sealant applied.

At tooth level, the decayed FPMs counted for 10.6% (n = 574) of the total number of teeth examined, while only 0.5% (n = 27) of the FPMs had fillings. There was obvious underuse of fissure sealants on the FPMs; 0.5% (n = 25). The remaining 4768 FPMs (88.4%) were sound (Table 1).

When comparing maxillary to mandibular FPMs (Table 1), the caries prevalence in the mandibular FPMs (14.4%), was significantly higher than the caries prevalence in the maxillary FPMs, 7.7% (p < 0.01). In contrast, there was no significant difference in the proportion of the presence of fissure sealants between maxillary and mandibular FPMs, 0.3% and 0.6% respectively (p > 0.05).

The results and comparisons between the three grades/age groups are presented in Table 2. The proportion of carious FPMs increased with age of the children significantly (p < 0.01). This was increased from 6.6% for the first grade students, to 14.1% for the third-grade students.

On the contrary, there was no significant difference in the proportion of the presence of fissure sealantsamong the three different grade/age groups (p > 0.05).

The results showed no association between age and fissure sealants use, as the results showed that fissure sealed FPMs were semi-constant between the different grade/age groups; 0.8%, 0.3%, and 0.4% for the first, second, and third grade, respectively. Additionally, there was no correlation between the proportion of decayed FPMs and the presence of fissure sealants (Table 2).

It was interesting to know that 56.6% of the FPMs were erupted in the first-grade boys (6 to 7 years old), 78.9% of FPMs have erupted in the second-grade boys (7 to 8 years old), and 86.4% of FPMs have erupted in the third-grade boys (8 to 9 years old).

## DISCUSSION

This survey investigated the caries prevalence and presence of fissure sealants on the FPMs exclusively, among 6 to 9 years old public school boys in Riyadh city, Kingdom of Saudi Arabia. Only the FPMs were evaluated since they are a key to the permanent dentition and have almost erupted in this age of children. Because of the exclusion of the partially erupted and unerupted FPMs, the further data analyses, and comparisons were conducted at tooth level instead of child level.

**Table 1:** **Table 1:** Maxillary versus mandibular FPMs caries and fissure sealants prevalence

		*Number of excluded teeth (partially erupted and unerupted)*		*Number of teeth examined*		*Decayed teeth n (%)*		*Filled teeth n (%)*		*Fissure sealed teeth n (%)*		*Sound teeth n (%)*	
Total		1982		5394		574 (10.6%)		27 (0.5%)		25 (0.5%)		4768 (88.4%)	
Maxillary		1074		2614		196 (7.5%)		6 (0.2%)		7 (0.3%)		2405 (92.0%)	
Mandibular		908		2780		378 (13.6%)		21 (0.8%)		18 (0.6%)		2363 (85.0%)	
Comparison		–		–		chi-squared = 52.7		chi-squared = 7.5		chi-squared = 2.7		chi-squared = 4.3	
						p < 0.01		p < 0.01		p > 0.05		p < 0.01	

**Table 2:** **Table 2:** Distribution and comparison of FPMs caries and fissure sealants prevalence based on age groups

				*Number of excluded teeth (partially erupted and unerupted)*		*Number of teeth examined*		*Decayed teeth n (%)*		*Filled teeth n (%)*		*Fissure sealed teeth n (%)*		*Sound teeth n (%)*	
Total				1982		5394		574 (10.6%)		27 (0.5%)		25 (0.5%)		4768 (88.4%)	
First grade (6-7 years old) No. of Children		= 672		1166		1522 (56.6%)		101 (6.6%)		3 (0.2%)		12 (0.8%)		1406 (92.4%)	
Second grade (7-8 years old) No. of Children		= 592		500		1868 (78.9%)		191 (10.2%)		1 (0.05%)		5 (0.3%)		1671 (89.5%)	
Third grade (8-9 years old) No. of Children		= 580		316		2004 (86.4%)		282 (14.1%)		23 (1.1%)		8 (0.4%)		1691 (84.4%)	
Comparison				–		–		Chi-squared = 50.8 p < 0.01		Chi-squared = 7.5 p < 0.01		Chi-squared = 2.7 p > 0.05		Chi-squared = 4.3 p < 0.01	

Several studies have investigated the prevalence of dental caries though not exclusively on FPMs in Saudi Arabia. To the best of our knowledge, this is the first report conducted in Riyadh, the capital city, regarding caries prevalence and the prevalence of fissure sealants on the FPMs specifically.

The results revealed a moderate proportionof identified untreated caries and a low proportion of subsequently placed restorations. Very low use of fissure sealants was noted for this population. None of the FPMs was recorded as missing due to caries, and this could be because of the young age of the groups involved, concluding that extraction of the FPMs due to caries was very rare in this population.

Comparing our results to previous global and national studies is quite difficult because of the differences in the studies’ design and the age of the targeted groups, in addition to other related variables.

In this study, the children who had at least one decayed or filled FPMs (i.e., caries prevalence) counted for 16.5% (n = 305 children). In other words, 83.5% of the children (n = 1539), did not have caries experience in their FPMs (i.e., caries free). This is considerably much lower than reported in some studies. Nationally, a study reported 67% of 9 years old children in Jeddah, Kingdom of Saudi Arabia, had caries in their FPMs.^8^ In another city; Abha, a study by Togoo et al.,^7^ who examined 7 to 10 years old children for the caries prevalence in their FPMs, it was reported that 66.4% of the children had carious FPMs. Globally, a study on Moroccan children aged 6 to 15 years old revealed that 77% of the children had caries prevalence in their FPMs.^20^ In Taiwan, a study by Warren et al.,^21^ on first-grade school children, aged 6 years old, resulted in 52% of children with caries prevalence in their FPMs. In contrast, in China, few reports had a wide variation in the caries prevalence among children aged in the range of 6 to 15 years old. In ascending order of their results, the caries prevalence in the FPMs was reported in 8.7% of 7 to 8 years old children,^22^ 26.5% in 7 to 9 years old,^23^ 47.5% in 7 to 9 years old,^24^ and 72% in 7 to 12 years old.^25^ The variation in caries prevalence worldwide and the involving of the older age of participants in some of the studies would be a possible explanation.

When analyzing the prevalence of fissure sealants on the FPMs, we verified in the current study that only 0.8% of the children (n = 15), had at least one fissure sealant applied.This result is very low and alarming of the underuse of fissure sealants in caries prevention among children.

In Germany, a study among 8 to 12 years old children, showed that 55.6% of children had at least one fissure sealant applied.^26^ Another study on adolescents aged 12 to 18 years old in Portugal reported that 59% of participants were with the presence of at least one fissure sealant on FPMs.^27^ In contrast, a very low fissure sealants prevalence was noted in 12 to 15 years old Greek adolescents (8%).^17^ The younger group of children in the current study would explain our low finding, although the fissure sealant is recommended to be placed as soon as the FPMs erupted.

At tooth level, the overall caries prevalence was in 11.1% of the FPMs, which indicates that 88.9% of the examined FPMs were free of caries. In particular, the caries prevalence was 6.6%, 10.2%, 14.1%, for first-grade children (6 to 7 years old), second-grade children (7 to 8 years old), and third-grade children (8 to 9 years old), respectively. Comparing these findings to other studies, showed a controversy view. A very early study by King et al.,^28^ revealed 10% carious FPMs among 7.4 years old children, and another recent study showed about 16% carious FPMs among 7 to 8 years old children in Poland.^29^ Our findings were in the range of both of these two surveys. However, the study conducted by Togoo et al.,^7^ in Abha, Saudi Arabia, showed a higher caries prevalence in the FPMs. In their study, the overall caries prevalence was 43.4% of the FPMs in a group of children aged 7 to 10 years old. In particular, the prevalence was 14%, 36%, and 57% among children aged 7 to 9 years old, respectively.

In this survey, the caries prevalence in the mandibular FPMs was significantly higher than the caries prevalence in the maxillary FPMs. This finding is in agreement with other studies.^7,23,30,31^ The reason expected behind this common finding, is the difference in the morphology and the earlier eruption time of mandibular compared to maxillary FPMs.^7^

The caries prevalence was statistically increased as the age increased. There is almost agreement in the literature that aging is accompanied with the increase of the caries prevalence of the FPMs among children, which is similar to our findings.^5,7,8,20,22-24^

Our finding of that 56.6% of FPMs were erupted in the first-grade boys (6 to 7 years old), agreed with a previous study which reported 50% of the FPMs were erupted by age of 7.4 years.^28^

It was clear that the carious process in the FPMs starts as soon as they erupt and can be clinically detected within 1 to 2 years.^8,30^ The results emphasize the need and importance of early prevention and educational programs which should be implemented even in an earlier age of the children.When considering fissure sealant application in children, it is recommended to be applied soon after tooth eruption.^30,32^

Considering the excellent results in caries reduction from other countries,^9,33-37^ where school-based fissure sealants programs were implemented, it is logical to recommend the introduction of the use of fissure sealants in schools-based or public preventive programs in Saudi Arabia.To plan and establish a national preventive program with fissure sealants in a community, it is essential to know the epidemiological pattern of caries and the current prevalence and use of fissure sealants. Additionally, the provision of fissure sealants is used as an indicator of the preventive care provided to children, on an individual or a public health basis.^11^

From the findings of this study, the use of fissure sealants in children was extremely low. This might be referred to lack of awareness of the public and that the dentists have not been convinced on the usefulness and effectiveness of sealants on caries prevention, or underusing them even knowing their effectiveness in caries prevention. This hypothesis is supported from the findings of another study conducted internationally aiming to investigate the dentist’s knowledge and opinion on sealant use.^17,38,39^ Further investigation of the associated factors behind the low use of fissure sealants nationally, both related to parents and dentists is recommended.

## CONCLUSION

- Caries prevalence in the FPMs was moderate but serious amongst this cohort of young students.The prevalence of caries in the FPMs increased with age. The caries prevalence in mandibular FPMs was higher than maxillary FPMs.
- Very low prevalence of fissure sealants on the FPMs, indicating the underuse of fissure sealants among schoolchildren examined in this study.
- School-based or national sealant programs should be promoted strongly and implemented as an effective preventive approach, complemented with oral health education.

## CLINICAL SIGNIFICANCE

Careful examination of the first permanent molars among children is mandatory because of their high caries susceptibility. It is important to increase public and dental professionals’ awareness of the effectiveness of fissure sealants and encouraging more placement.
